# Differential diagnosis of auditory hallucinations in teenagers. Assessment and difficulties: Case report of a 13 year old patient

**DOI:** 10.1192/j.eurpsy.2021.625

**Published:** 2021-08-13

**Authors:** C. Pastor Jordá, P. Carroll, D. Bender, R. Tumuluru

**Affiliations:** 1 Child And Adolescent Psychiatry, Alicia Koplowitz Foundation, Madrid, Spain; 2 Child And Adolescent Psychiatry, UPMC Western Psychiatric Hospital, Pittsburgh, United States of America

**Keywords:** Hallucinations, Teenagers

## Abstract

**Introduction:**

Learning from a case of a 13 year old patient with auditory hallucinations for 2 months, admitted to the hospital due to suicidal ideation. Her mother had been diagnosed with Lupus and OCD. Her mood had been low for several months, probable mild intellectual disability.

**Objectives:**

Learn how to assess auditory hallucinations and possible new onset psychotic symptoms in teenagers. Learn about different levels of care involved. Discuss differential diagnosis and future directions and treatment.

**Methods:**

Description of the case. Differential diagnosis: Obsessive compulsive disorder, Major depressive disorder with Psychotic features, schizophrenia spectrum disorder, epilepsy or other neurologic disease, autoimmune disease, post-traumatic stress disorder… Tests and consults conducted by Neurology team Psychopharmacology description.

**Results:**

Differential diagnosis: Obsessive compulsive disorder, Major depressive disorder with Psychotic features, schizophrenia spectrum disorder, epilepsy, autoimmune diseases like Lupus, post-traumatic stress disorder etc. Video EEG: normal. Brain MRI: normal Blood work unremarkable with positive ANA (titer 1:80). Work up, including lumbar puncture with autoimmune encephalitis and MS panels was negative. Psychopharmacology: Fluoxetine up to 40mg, and Aripiprazol up to 20mg without a good response. Possible sexual trauma was disclosed in a second hospitalization, months later.

**Conclusions:**

Recommendation of assessing new onset of psychotic symptoms in detail to get a good diagnosis. Psychotic symptoms in young teenagers may occur as part of different presentations and it is important to provide a good follow up of the patient in order to provide the most accurate treatment.

**Conflict of interest:**

Alicia Koplowitz Foundation

